# Conjugation of Benzylvanillin and Benzimidazole Structure Improves DNA Binding with Enhanced Antileukemic Properties

**DOI:** 10.1371/journal.pone.0080983

**Published:** 2013-11-15

**Authors:** Zena A. Al-Mudaris, Aman S. A. Majid, Dan Ji, Ban A. Al-Mudarris, Shih-Hsun Chen, Po-Huang Liang, Hasnah Osman, Shah Kamal Khan Jamal Din, Amin M. S. Abdul Majid

**Affiliations:** 1 School of Pharmaceutical Sciences, University Sains Malaysia, Minden, Penang, Malaysia; 2 Advanced Medical and Dental Institute, Universiti Sains Malaysia, Bandar Putra Bertam, Penang, Malaysia; 3 Key Laboratory of Visual Damage and Regeneration & Restoration of Chongqing, Southwest Eye Hospital, Southwest Hospital, The Third Military Medical University, Chongqing, P.R.China; 4 College of Dentistry, Ajman University, Ajman, UAE; 5 Department of Biological Science and Technology, National Chiao Tung University, Hsin-Chu, Taiwan; 6 Institute of Biological Chemistry, Academia Sinica, Taipei, Taiwan; 7 School of Chemical Sciences, University Sains Malaysia, Minden, Penang, Malaysia; 8 OMF Surgery, Hospital Sultanah Bhiyah, Alor Setar, Kedah, Malaysia; Aligarh Muslim University, India

## Abstract

Benzyl-o-vanillin and benzimidazole nucleus serve as important pharmacophore in drug discovery. The benzyl vanillin (2-(benzyloxy)-3-methoxybenzaldehyde) compound shows anti-proliferative activity in HL60 leukemia cancer cells and can effect cell cycle progression at G2/M phase. Its apoptosis activity was due to disruption of mitochondrial functioning. In this study, we have studied a series of compounds consisting of benzyl vanillin and benzimidazole structures. We hypothesize that by fusing these two structures we can produce compounds that have better anticancer activity with improved specificity particularly towards the leukemia cell line. Here we explored the anticancer activity of three compounds namely 2-(2-benzyloxy-3-methoxyphenyl)-1H-benzimidazole, 2MP, N-1-(2-benzyloxy-3-methoxybenzyl)-2-(2-benzyloxy-3-methoxyphenyl)-1H-benzimidazole, 2XP, and (R) and (S)-1-(2-benzyloxy-3-methoxyphenyl)-2, 2, 2-trichloroethyl benzenesulfonate, 3BS and compared their activity to 2-benzyloxy-3-methoxybenzaldehyde, (Bn1), the parent compound. 2XP and 3BS induces cell death of U937 leukemic cell line through DNA fragmentation that lead to the intrinsic caspase 9 activation. DNA binding study primarily by the equilibrium binding titration assay followed by the Viscosity study reveal the DNA binding through groove region with intrinsic binding constant 7.39 µM/bp and 6.86 µM/bp for 3BS and 2XP respectively. 2XP and 3BS showed strong DNA binding activity by the UV titration method with the computational drug modeling showed that both 2XP and 3BS failed to form any electrostatic linkages except via hydrophobic interaction through the minor groove region of the nucleic acid. The benzylvanillin alone (Bn1) has weak anticancer activity even after it was combined with the benzimidazole (2MP), but after addition of another benzylvanillin structure (2XP), stronger activity was observed. Also, the combination of benzylvanillin with benzenesulfonate (3BS) significantly improved the anticancer activity of Bn1. The present study provides a new insight of benzyl vanillin derivatives as potential anti-leukemic agent.

## Introduction

Novel benzaldehyde compounds containing benzimidazole have been reported to have promising chemotherapeutic potential that has an alternate mechanism of action than methoxybenzaldehyde based drugs [1]. It has also been found that benzaldehyde compounds can cause apoptosis in cancer cell lines that make them exceedingly important in the treatment and prevention of the disease [2,3]. DNA minor groove binders (MGBs) is a novel family of antitumor agents that have entered clinical trials [4]. During the last decade, many synthetic MGBs have been reported, including analogues and conjugates of naturally occurring minor groove-binding agents, such as distamycin (Dst), netropsin (Net), CC-1065, anthramycin (Atm), and Hoechst 33258 [5]. Hoechst 33258, a fluorescent reagent with a head-to-tail bis-benzimidazole structure, was initially found to be active against L1210 murine leukemia [6]. Due to the synthetic accessibility and high binding affinity of Hoechst 33258, several investigators had utilized the pharmacophore-like benzimidazole motif derived from Hoechst 33258 [7,8,9,10]. In recent years many efforts have been made to develop probes that target specific DNA sequences for a variety of uses from diagnostic to therapeutic application [4,6,11]. 

Natural products and synthetic organic cations that bind specifically and selectively to the DNA minor groove can do so by a combination of ionic, hydrophobic and hydrogen bonding interaction [12,13,14]. For this purpose small molecules have to meet classical structural criteria for optimum DNA fit and interaction which include, crescent shaped structure that complements the helical DNA minor groove, recognition units (H-bond donors and acceptors) on the side of the molecule facing DNA, cationic centre at terminals of the molecules to enhance electrostatic interactions, extended unfused heterocyclic structure to allow optimization of the compound for DNA minor groove interactions [15,16,17].

Benzimidazoles are weak mutagens acting through base substitutions. They are incorporated into nucleic acids [18] and can be regarded as a structural analogue of purine. This feature attracted early attention and indeed it has been found that a benzimidazole-caused growth inhibition can be reversed by purines [19]. Later, Novick [20] and Szybalski [21] found benzimidazole to be mutagenic in the E. coli systems. With the Salmonella typhimurium strains of Ames [22], it was possible to interpret benzimidazole mutagenicity as a base substitution [23]. Not unexpectedly, some researchers found the incorporation of benzimidazole into the nucleic acids of E. coli [24]. Furthermore a number of other investigators found that many benzimidazole basic structures have antioxidant activity hence are useful in minimizing lipid peroxidation [25].

Among the ligands employed for drug design, benzimidazole nucleus serves as an important pharmacophore in drug discovery [26]. The benzimidazoles are a subject of interest to medicinal chemistry [27] and are useful subunits for the development of molecules for pharmaceutical or biological application [28]. They are found in many bioactive compounds from natural and synthetic sources [29,30].

Another pharmaceutically interesting compound is the benzyl vanilline (2-(benzyloxy)-3-methoxybenzaldehyde). This compound shows anti-proliferative activity in HL60 leukemia cancer cells and can effect cell cycle progression at G2/M phase [31]. Its apoptosis activity was due to disruption of mitochondrial functioning [32].

In this study, we have synthesized a series of compounds consisting of benzyl vanilline and benzimidazole structures. We hypothesize that by fusing these two structures we can produce compounds that have better anticancer activity with improved specificity particularly towards the leukemia cell line. 

**Figure 1:**
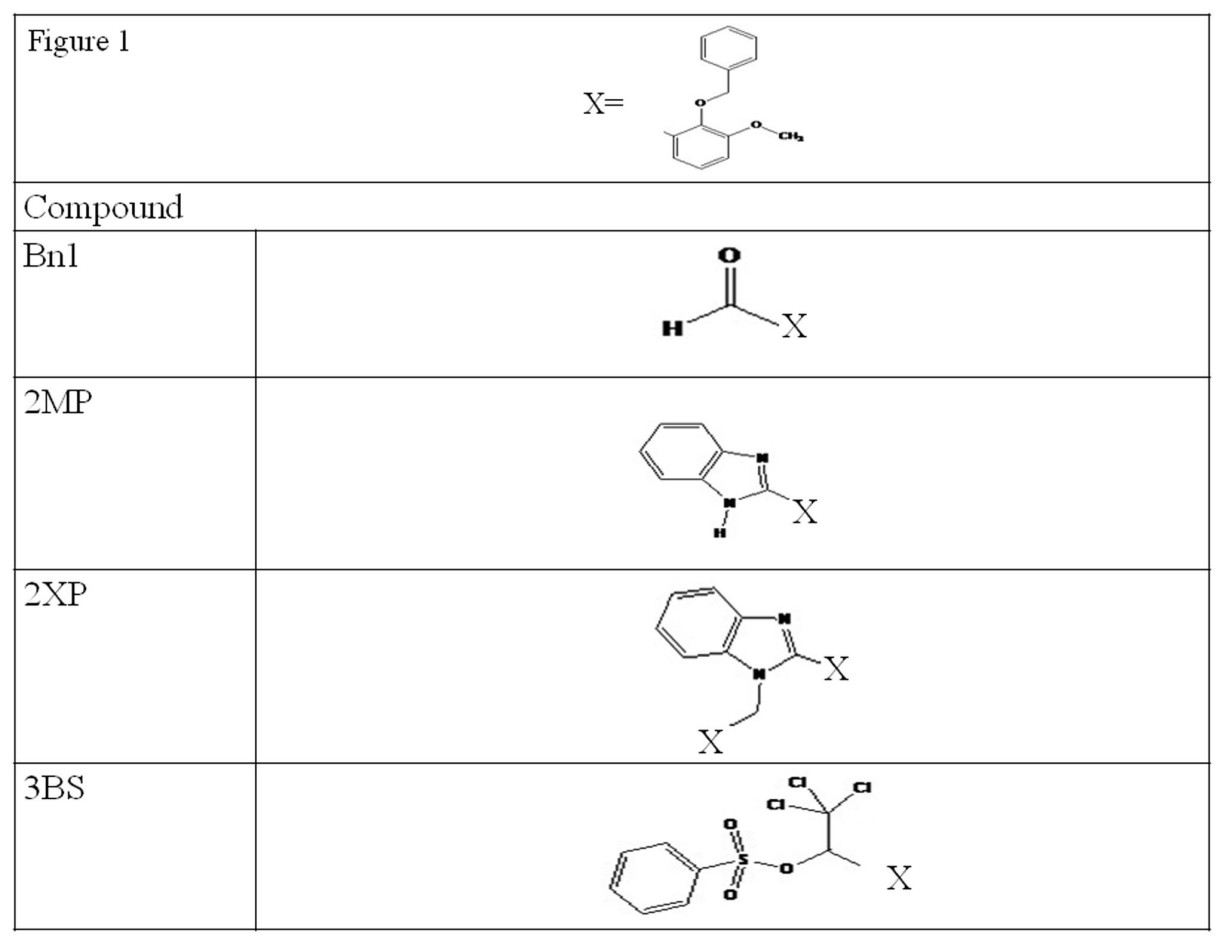
Chemical structures of Bn1, 2MP, 2XP and 3BS.

The four compounds investigated in this work are 2-benzyloxy-3-methoxybenzaldehyde, Bn1, 2-(2-benzyloxy-3-methoxyphenyl)-1H-benzimidazole, 2MP, N-1-(2-benzyloxy-3-methoxybenzyl)-2-(2-benzyloxy-3-methoxyphenyl)-1H-benzimidazole, 2XP, and (R) and (S)-1-(2-benzyloxy-3-methoxyphenyl)-2, 2, 2-trichloroethyl benzenesulfonate, 3BS. The crystal structures of these compounds have been recently published [32,33,34,35]. Figure 1 shows the chemical structures of these compounds.

## Materials and Methods

### Cytotoxicity assay

In brief, cells were seeded at 2-5×10^4^ cell density per well for each 96-well plates in 180µl medium for attached cells (MDA-MB231, MCF10, HT29 and FHC from ATCC, USA) and 100µl medium for suspended cells, U937 from ATCC, USA. Then the compounds were added separately into the well using the stock solution to make the final concentration 200, 100, 50, 25, 12.5, 6.25, 3.125 and 1.562µM. The untreated cells received only 1% DMSO (Sigma) as a negative control and Mechlorethamine (Sigma) and Tamoxifen (Sigma) as positive control. All cells were treated for 48h. The experiment was repeated three times with four replicates for each concentration. At the end of the treatment period (48h), 20µl of XTT solution (Sigma) was added to each well. After 4h incubation at 37°C, the optical density was recorded using a plate reader (Multiskan Ascent) at 490nm for absorbance and 650nm as reference filter.

### Apoptosis assay

Briefly, U937 cell line were plated in a 96 well-plate at 5 ×10^5^ cell/ml and treated with 50µM in triplicate for each compound. After 1, 3, 5, 7, 12 and 24 hours, 100µL of the Caspase-3/7 reagent (Promega) was added to each compounds. After one hour incubation, the luminescence emission spectra were measured for the samples. Betulinic acid (Sigma) was used as a positive control at 50µM concentration and 1% DMSO was used as a negative control. Same protocol was also followed using caspases-8 and -9 (Promega).

### Cell morphology study

U937 cell line was treated with different concentration of 2XP and 3BS separately, 1% DMSO and betulinic acid was used as a negative and positive control respectively. After 48 hours of the incubation, pictures of the cells were taken with the aid of an inverted microscope equipped with an imaging system (Olympus, Japan).

### DNA fragmentation assay

The DNA extraction and purification was carried out according to manufacturers protocol (Promega, USA). In brief, 2.5ml of U937 was seeded at 1x10^4^ - 5x10^6^ cells per well. The cells were treated with different concentration (20, 60, 100µM) of Bn1, 2MP, 2XP and 3BS for 48 hours followed by washing with 1 x PBS (Sigma). 150µl of lysis buffer (Promega) was added to the solution. Each lysate mixture was then transferred to a mini-column (Promega) and then centrifugated at 13000g for 3min. The remaining solution was washed with 95% ethanol followed by further centrifugation for 1 min. 250µl of neuclease free water (Promega) was added to the solution for 2 min and this was centrifuged for a further 1 min to produce the purified DNA. 30µl of the purified DNA product was loaded onto agarose gel (1.2%) with 5% ethidium bromide with one well containing untreated DNA. 5µl of 6X loading dye was added to each well, mixed thoroughly. The gel electrophoresis was run at 100V for 2 hours. The gel slab was then analysed with a gel imaging system (GelDoc Reader, BioRad).

### Equilibrium binding titration

The procedure was performed according to previous published work [36]. Briefly, 10µL solution containing 1mM of each compound was diluted to 500 µL using the standard buffer (Standard buffer solution containing 0.15M NaCl (Sigma), 0.50mM MgCl2 (Sigma), and 10mM phosphate buffer (pH=7.3) [37]). UV absorbance was measured at 200-600 nm wavelengths after each addition of calf thymus DNA (Sigma). The volumes of DNA added to each compound solution were 0 µL, 2 µL, 4 µL, 8 µL, 10 µL, 15 µL, 20 µL, 25 µL, 30 µL, 40 µL, 50 µL 60 µL and 70 µL to give effective DNA concentration of 0, 0.36, 0.72, 1.44, 1.08, 2.7, 3.60, 4.5, 5.4, 7.2, 9, 10.8, 12.6 and 14.4 µM respectively. The DNA was added until no apparent decrease in absorption was observed The UV absorbance values were measured using a UV spectrometer (Perkin Elmer Lambda 45). The drug binding fraction α, and the equilibrium distribution at each titration position is calculated according to the following formula: Α=*Cb*/*C*=*(*1-*Cf*/*C*)=*(A*_*f*_^0^-*A*)/*(A*_*f*_^0^-*A*_*b*_^0^). A_f_^0^ and A_b_^0^ are the measured absorptions for the free and fully bound drug at the monitoring wavelength. *r=α.C/C*_*DNA*_ and *C*_*f*_=*(*1-α).*C*, where CDNA is the total concentration of DNA or oligonucleotide titrant at each point. The binding constant value K, was determined by plotting a scatchard plot of r/Cf vs r [36].

### Viscosity measurement assay

Viscometer experiments were performed using an Ubbelohde viscometer (Cannon, USA). The temperature was maintained at room temperature (25°C) with the aid of a water bath. 600 µl of 150µg/ml calf thymus DNA solution was placed in the viscometer and allowed to pass through the small capillary tube. The time taken for the sample to pass through was measured by using a digital stop watch. This procedure was repeated but with the addition of varying concentration of 2XP and 3BS to the calf thymus DNA. A volume of 12, 24, 48 and 96µL containing 60mM of the individual compounds were added to 3ml of the 150µg/ml calf thymus DNA to give compound-DNA ratio of 0.8, 1.6, 3.2 and 6.4. Ethedium bromide (Sigma) and Hoechst 33258 (Sigma) were used as positive control representing intercalation and minor groove binding compounds respectively. The time required for each concentration to pass was recorded. Each measurement was repeated 5 times.

The viscosity can be calculated though the following equation which is derived from Poiseuille's law [38]: *Ƞsp = Ƞr -1= t — t*_*o*_ */ t*_*o*_. *Ƞ*_sp_ is the specific viscosity, t_o_ is the time needed for elution for the buffer alone and t is the elution time needed for the solution. 

### Computational docking study

The computer modeling of the four compounds binding to DNA was done using the ParDOCK protocol in web-enabled software at www.scfbio-iitd.res.in/dock [39].

### Statistical analysis

Statistical analysis was performed with GraphPad Prism (Version 5.00) software (GraphPad Software, Inc., San Diego, CA, USA). 

## Results

### Cytotoxicity studies

The results of the cytotoxicity studies for Bn1, 2MP, 2XP and 3BS are presented in Table 1. The cytotoxic activity was measured in terms of their IC50 values, the drug concentration at which cell growth is reduced by half. Lower IC50 indicates stronger cytotoxicity of a compound. The results show that 3BS has the highest level of cytotoxic activity towards U937 followed by 2XP, 2MP and Bn1 in reducing order of activity. 

**Table 1:** Half maximal inhibitory concentration (IC_50_) values of different compounds against the five cancer cell lines.

**Cell lines**	**Bn1**	**2MP**	**2XP**	**3BS**	**Tamoxifen**	**Mechlorethamine**
**FHC**	40.47±1.4	29.85±2.01	14.58±1.6	4.54±1.21	-	2.33±1.01
**HT29**	57.39±1.17	45.30±1.2	42.40±1.12	24.37±1.07	-	49.23±1.07
**MCF10**	80.18±0.73	71.73±1.42	83.13±1.27	40.83±2.12	10.45±0.48	-
**MDA-MB 231**	566.5±0.99	30.60±1.97	39.6±1.66	1780±0.71	1.32±0.61	-
**U937**	51.57±1.07	67.93±1.07	12.60±1.1	10.94±1.05	-	4.89±1.05

### Apoptosis studies

**Figure 2:**
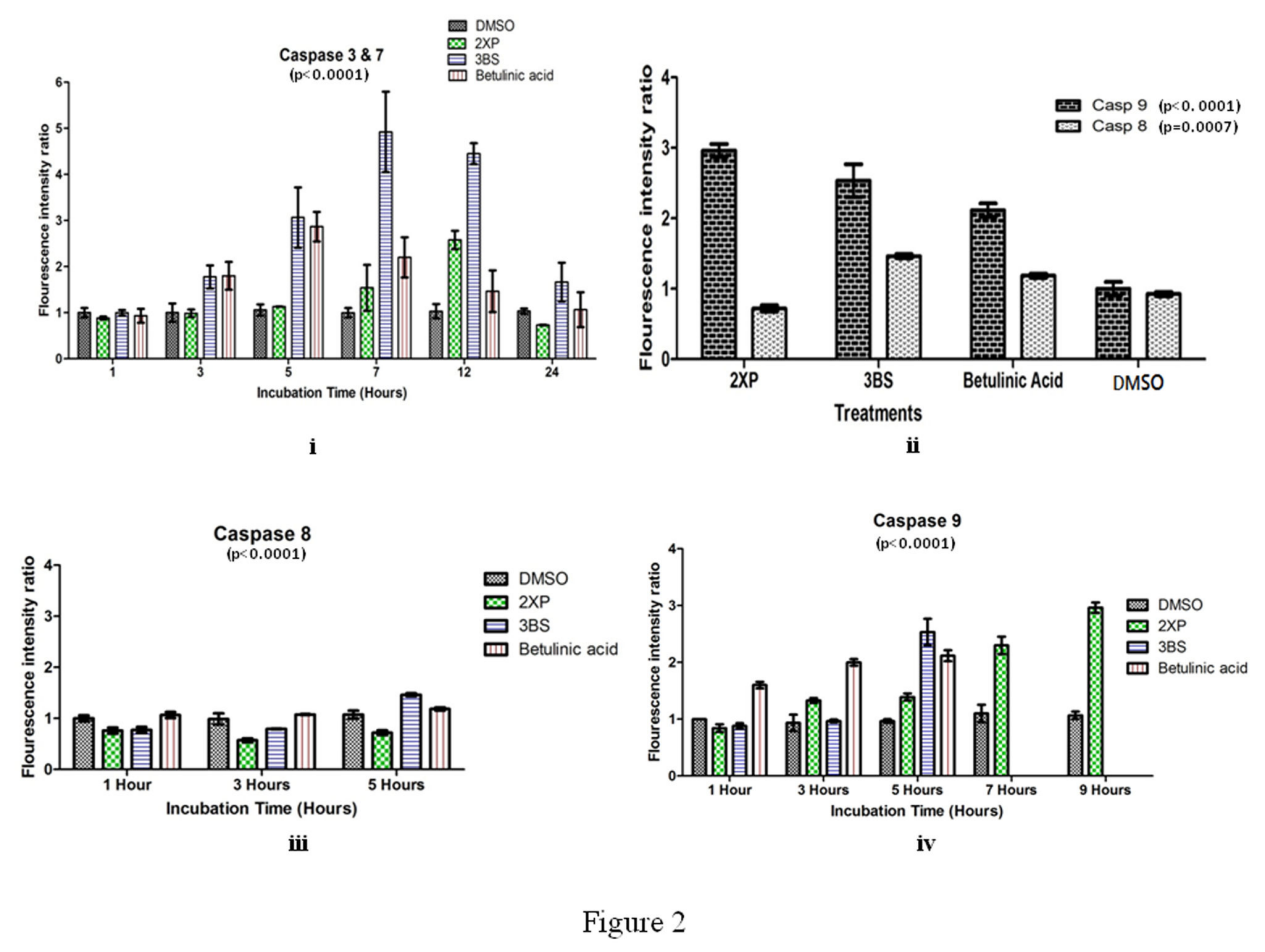
The activity on caspase activation enzymes after 2XP, 3BS and betulinic acid exposure. (i) The fluorescence intensity of caspase enzymes ratio on U937 cells after exposure to 2XP, 3BS and betulinic acid (as a positive control) at different time intervals. caspase 3&7 with significant difference between the treatments and the control group at 5hrs, 7hrs and 12hrs incubation period. p<0.01, (ii) comparison caspase 8 & 9 during the maximum peak period with significant difference between the caspases, p<0.0001 and within the treatment, p=0.047, (iii) caspase 8 with significant difference between the treatments and the control (DMSO) at 3hrs and 5hrs of incubation period. p<0.01, (iv) caspase 9 with significant difference among the treatments at 3hrs, 5hrs, 7hrs and 9hrs of incubation period. p<0.01. Note: The results for 3BS and betulinic acid are not displayed for 7hrs and 9 hrs as their respective caspase 3 activation occurs at 5 hours.

The studies so far clearly show the cytotoxicity on leukemia cell line for 2XP and 3BS. To better understand the mechanism of action that armed these two compounds with high cytotoxicity level, we studied their activity on caspase activation enzymes namely casapase 3&7 (Figure 2(i)). Following that, we studied their effect on the caspase 8 and caspase 9-activation (Figure 2(ii), 2(iii) and 2(iv)).

In caspase 3&7 activity, there was a significant difference observed among all the incubation time, p<0.0001, and also among the treatment compounds, p<0.001 while in caspase 8 activity, there was a significant difference among the incubation time, p<0.0001, and also among the treatments, p=0.0007 and in caspase 9 activity, there was a significant difference among the incubation time, p<0.0001, and also among the treatments, p<0. 0001.

### Cell morphology study

**Figure 3:**
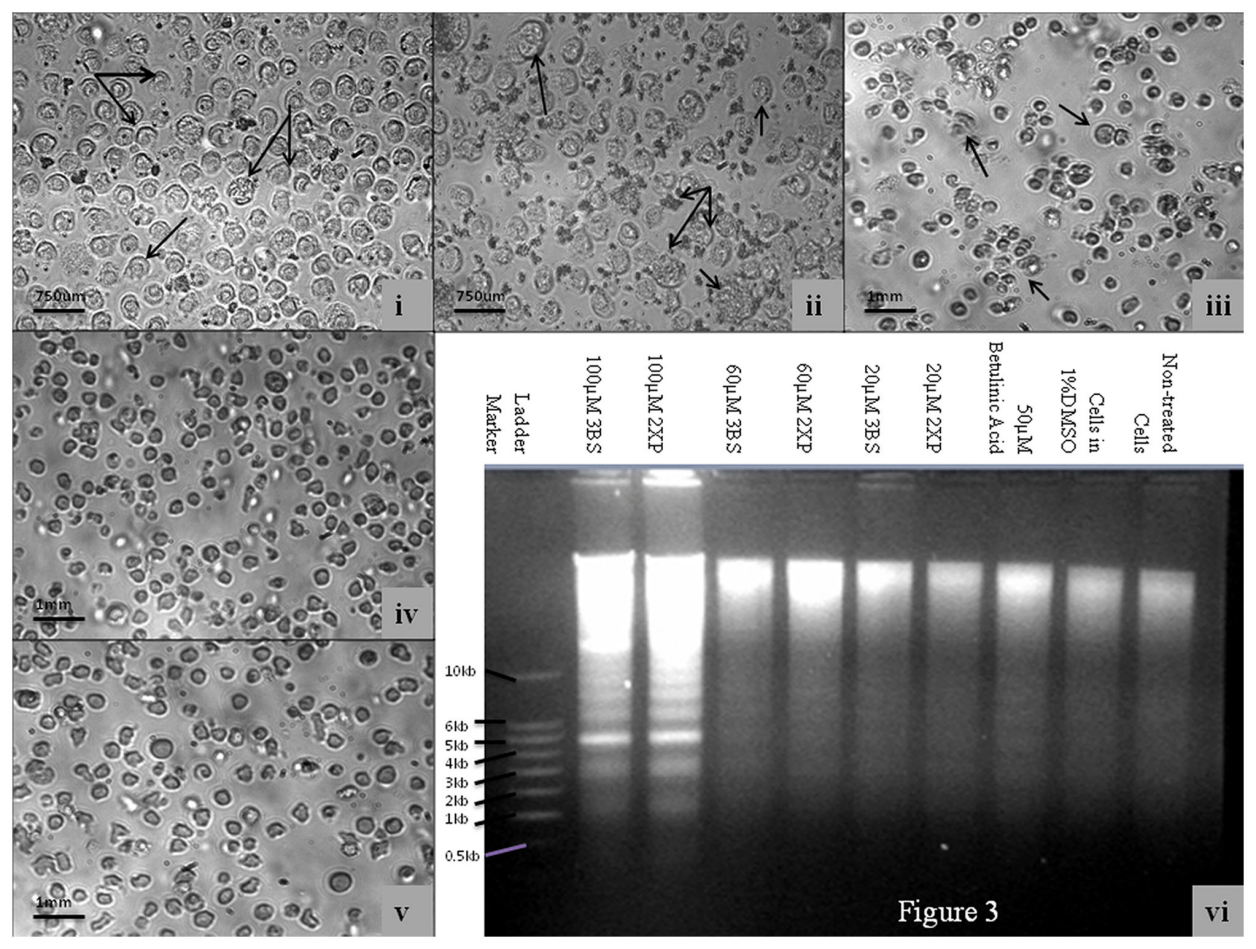
Cell Morphology of a Human Leukemic cancer cell lines (U937) after different treatment. (i) 50μM 2XP, (ii) 50μM 3BS, (iii) 50μM betulinic acid, (iv) 1% DMSO and (v) medium alone. Arrows indicate area of apoptosis. Figure 3(vi) shows the DNA fragmentation of nucleic acid extracted from U937 after treating with 20, 60 and 100 µM of 2XP and 3BS as well as betulinic acid (positive control) and DMSO (negative control).

Figures 3(i), 3(ii), 3(iii), 3(iv) and 3(v) show the U937 cell morphology after treatment with 2XP, 3BS, butilic acid, 1%DMSO and non treated cells respectively for 48 hours and the DNA fragmentation activity is shown on Figure 3(vi). The apoptotic cells shown clearly as crescent shaped (as indicated by arrows) with the early apoptotic U937 cells showing chromatin condensation typical of budding fragmentation and total nuclear fragmentation in late apoptotic U937 cells: many nuclear fragments are spread in the cytoplasm. Chromatin condensation and nuclear fragmentation by cleavage in early apoptotic U937 cells and nuclear fragments at the end of the fragmentation resulting from cleavage (late stage) are forming a cluster.

### DNA Fragmentation Assay

The extraction and purification were done according to the supplier’s protocol (Promega, USA). For the gel Electrophoresis, the procedure was performed according to previous published work [40,41]. To prepare the samples by putting 30µl of purified DNA (treated one) with 5µl of 6X loading dye. For the ladder marker, 10Kb, and untreated DNA, 5µl was taken then added 1µl of 6X loading dye. Mix the mixture by pipetting thoroughly. Loading the samples on each comb separately and run the electrophoresis on 1000V for 2 hours then read the gel on GelDoc Reader by BioRad.

### DNA Binding studies

#### Equilibrium binding titration

**Figure 4:**
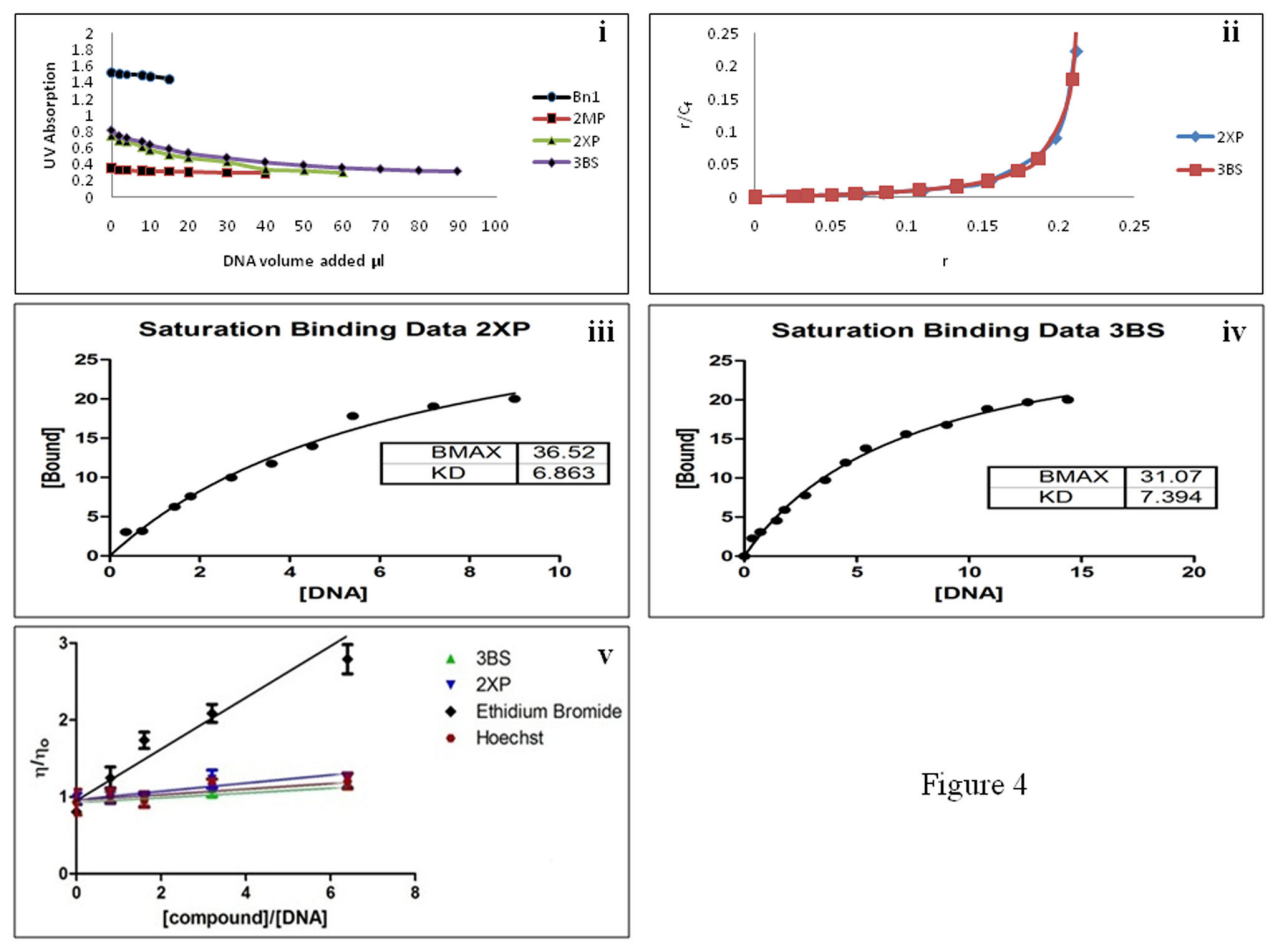
UV spectroscopy and viscometery analyses on the different compounds. (i) the UV Absorption at λmax for Bn1, 2MP, 2XP and 3BS during the addition of DNA, (ii) Scatchard of saturation binding of 2XP and 3BS. Analysis of absorbance data at 221 and 216 for binding of 2XP and 3BS to calf thymus DNA respectively, (iii) the saturation curve of DNA binding to 2XP and (iv) the saturation curve of DNA binding to 3BS (v) the effect of 3BS, 2XP, EtBr (positive control as DNA intercalator) and Hoechst (positive control as groove binder) on DNA viscosity.

Spectral results of DNA binding with Bn1, 2MP, 2XP and 3BS are presented in Figure S1(i), S1(ii), S1(iii) and S1(iv), respectively which show the various absorption spectra for all compounds before and after mixing with the calf thymus DNA. Figure S1(i) and S1(ii) show no bathochomic shift after addition of DNA to Bn1 and 2MP respectively. 2XP and 3BS spectra show significant shift and decrease in the UV absorbance spectrum of the compounds following the addition of DNA as seen in Figure S1(iii) (the bathochromic shift in value of λmax, from 221 to 226 nm, and the decrease in absorption at λmax (hypochromic shift) during addition of DNA to 2XP solution) and Figure S1(iv) (the bathochromic shift in value of λmax for 3BS, from 216 to 222 nm, and the decrease in absorption at λmax (hypochromic shift) during addition of DNA to 3BS solution). Scatchard equation was applied to find the intrinsic coefficient of each compound towards the DNA and their strength of binding, Figure 4(ii), 4(iii) and 4(iv).

#### Viscosity measurement Assay

The results of the viscosity experiments shows that 2XP and 3BS do not cause significant increase to the DNA solution viscosity compared to the well established intercalator EtBr which acts as the control for this experiment (Figure 4(v)). Hoechst 33258 reagent is used as a positive control to represent a minor groove binder. The viscosity reading for the Hoechst 33258 compound is similar to that of 2XP and 3BS.

#### Computational docking studies

**Figure 5:**
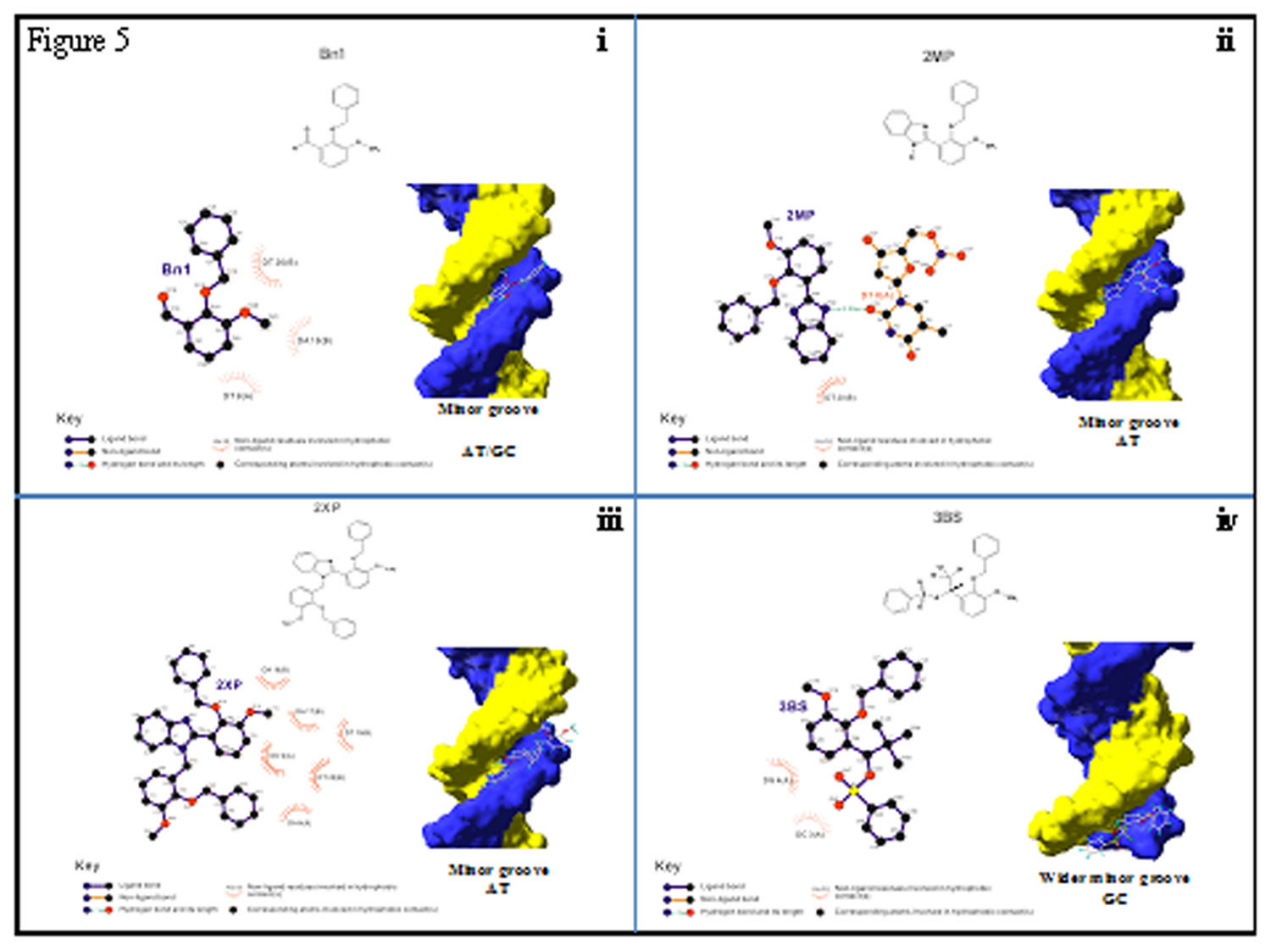
The computational modeling studies on the four compounds. The drug modeling for, i. Bn1, ii. 2MP, iii. 2XP and iv. 3BS respectively with DNA, show the binding site of each compound with their respective DNA.

The modeling results of the four compounds when docked against (CGCGAATTCGCG)_2_ sequence are shown in Figure 5. In Figure 5(i), the Bn1 molecule appears to bind in the minor groove of the nucleic acid. Here we find Bn1 binds via hydrophobic interaction mainly at C12, C16, C17 and C29 of Bn1 with thymine (T8) of the A strand and with adenine (A18) and thymine (T20) of the B strand. The energy of binding was calculated to be -7.3kcal/mol. Bn1 also show a clear electrostatic hydrogen bond linkage between the oxygen (O19) of Bn1 and the nitrogen (N2) of the guanine in the DNA strand and hydrophobic interaction with the cytosine and guanine of the DNA strand. The calculated free energy of binding was found to be between -7.2 to -8.0kcal/mol.

Figure 5(ii) shows the modeling results of 2MP docking, where the 2MP molecule appears to bind in the narrow minor groove region of the DNA. The result also shows clear electrostatic hydrogen bond linkages between the nitrogen (N1) of the benzimidazole structure of 2MP and the oxygen (O2) of the thymine (T8) on the A strand of the nucleic acid. There was also a hydrophobic interaction between the aromatic carbons (C38 and C42) of the benzimidazole with the thymine (T20) of the B strand of the DNA. The energy of binding was calculated to be -7.9kcal/mole. 

The computational modeling studies on 2XP docking, Figure 5(iii), shows lack of any chemical linkages between the 2XP structure and the nucleic acid. However, the data also shows that the compounds appears to reside snuggly in the narrow minor groove region of the deoxyribonucleotide particularly within the AT sequences. The result also shows significant hydrophobic contact between the aromatic and to lesser extend the non-aromatic carbons (C16, C19, C25, C27, C29, C43, C61, C64, C69 and C73) of benzyl-o-vanillin in 2XP structure and the adenines (A6) of A strand and A17, A18, T19 and T20 of B strand and to a lesser extent the cytosine (C9) of A strand of the DNA. The energy of binding was calculated to be -9.2kcal/mole.

Figure 5(iv) shows the modeling results of 3BS when docked against DNA. The 3BS molecule appears to reside in the wider minor groove section of the nucleic acid. The result of this study shows lack of any apparent linkages between the molecules however a clear hydrophobic contact can be observed between the aromatic and non-aromatic carbon (C12, C16, C27 and C29) of the benzyl-o-vanilin in 3BS structure and the guanine (G4) and cytosine (C4) of the A strand of the DNA. The energy of binding was found to be -7.2kcal/mole. 

## Discussion

Preliminary cytotoxicity studies on leukemia cell lines using 2-(benzyloxy)-3-methoxybenzaldehyde (benzyl vanilline) have been found to be moderately cytotoxic [31]. In order to improve the cytotoxic response, a variety of benzylvanillin structural analogues have been designed and synthesized so as to understand the structural features that are important to enhance the anticancer activity of this compound [33,34,35].

Bn1, 2MP and 3BS are three benzyloxy-3-methoxyphenyl base structural analogues, which differ in their side-chains. Bn1 has an aldehyde moiety and 2MP has a benzimidazole structure attached to its parent benzyloxy-3-methoxyphenyl base while 3BS has a trichloroethyl benzenesulfonate unit instead. The structure of 2XP differs to 2MP whereby an additional benzyloxy-3-methoxyphenyl structure is added to its present benzimidazole moiety, Figure 1. 

The results of this study reveal that the four compounds Bn1, 2MP, 2XP and 3BS differs significantly in their cytotoxic response towards the various cancer cell lines tested (see Table 1). 3BS were found to be most cytotoxic towards FHC normal colon cells (4.5µM) followed by U973 leukemia cell line (10.9µM), HT29 colon cancer cells (24.3µM), MCF10 breast cancer cells (40.8µM) and MDA-MB231(1780µM). When compared to the cytotoxic activity of 2XP, the compound gave the strongest cytotoxic response towards U973 leukemia cell line (12.6µM) followed by FHC (14.6µM), MDA-MB231 (39.6µM), HT29 (42.4µM) and MCF10 (83.1µM) in decreasing order of activity. The higher cytotoxic activity towards the normal colon cells (FHC) as opposed to the colon cancer cell lines HT29 is due to the ability of the latter to thwart DNA damage by up-regulating bcl-2 which cause marked reduction in the amount of compounds that can penetrate the cell membrane [42].

Bn1 and 2MP were only moderately cytotoxic towards all the cell lines tested. However, it is clearly obvious that the addition of the benzimidazole structure to the parent benzyloxy-3-methoxyphenyl caused a marked improvement in the cytotoxicity activity (except for U937 leukemia cells). It appears the addition of benzimidazole substructure also increased extent of DNA binding as shown in the DNA titration studies (see figure S1 and 4(i)). Both the hypochromic and bathochromic effect improved significantly in 2MP when compared to Bn1. Previous studies have shown that the NH group of the benzimidazole can easily form bifurcated hydrogen bonds with the thymine O2 or adenine N3 in the minor groove region of the nucleic acid [43]. It may perhaps be that this new compound 2MP, may be interacting with the AT rich DNA narrow minor groove region or even in the wider minor groove giving rise to the improvement in its cytotoxic response. The differences in cytotoxic response observed in the various cell lines is probably due to the nature of the cells which differ significantly in their growth rate [44]. Of course, it is also possible that the variation could also be due to interaction with key molecular targets that may be more pronounced in a particular cell type. 

The strong cytotoxic response towards the leukemia cells by 3BS and 2XP, encouraged us to analyze further the mechanisms of action of these compounds towards the leukemia cancer cell line. 

2XP and 3BS were found to cause marked induction of the caspases that led to the induction of apoptosis. Figure 2(i) clearly shows that 3BS was able to induce stronger apoptotic response than the positive control, betulinic acid. The level of caspase induction by 2XP was found to be less than 3BS. The level of caspase 9 activation, Figure 2(iv), for 2XP is significantly higher than 3BS, which also show a minor activation on caspase 8, Figure 2(iii). Both 2XP and 3BS are also significantly stronger caspase inducers than the negative control DMSO (p<0.0001). Because apoptotic response that leads to caspase 9 activation is mainly attributed by DNA damage and mitochondrial insult, we believe that 2XP and 3BS may hinder the leukemia cell survival by damaging its DNA and mitochondrial functioning. The result of the DNA fragmentation study firmly suggests that these two compounds have indeed triggered apoptosis response (see Figure 3(vi)). Evidence of cytotoxic activity of the compounds cited are illustrated in figure 3(i), 3(ii) and 3(iii) which show clear indication of apoptotic cell death based on the cell morphology.

In order to understand how these two compounds interacted with the DNA, a series DNA binding studies were carried out. The UV spectroscopy analysis on the interaction between 2XP and 3BS with calf thymus and leukemia cells DNA showed good binding activity. UV spectroscopy and viscometery analyses clearly indicate that 3BS and 2XP can bind to the DNA with the former exceeding the latter in its binding strength. By calculating Kd binding constant for both compounds, it was found that the Kd value for 3BS was 7.39µM per base pair, where else the Kd for 2XP was found to be 6.86µM/bp. This result of DNA binding is consistent with the findings in the viscometry studies, confirming the binding for 2XP and 3BS with DNA. In the viscometry analysis, it was found that the DNA viscosity increased significantly when the amount of 2XP and 3BS was increased (see Figure 4(v)). The extent of increase in viscosity was appreciably lower than ethidium bromide and was more closely related to Hoechst. The viscosity study is based on the principle that intercalating compounds can cause stabilization of the DNA hence making it more rigid and viscous [45,46]. If 2XP and 3BS interacts with the nucleic acid via intercalation, a significant increase in DNA viscosity would have been observed. Instead when compared to ethidium bromide, a well established intercalator [47,48,49], the extent of viscosity was significantly lower as was similar to the well recognized minor groove binder Hoechst. This suggests that 2XP and 3BS are poor DNA intercalators and may in fact bind in the groove region. The viscosity studies hints that the DNA interaction that could have possibly taken place may perhaps be via minor groove binding for 2XP and DNA alkylation for 3BS. It is also possible that 3BS may also interact covalently in the minor groove region similar to some alkylating minor groove binders [50,51,52]. Monofunctional alkylation can occur on the most nucleophilic sites on the DNA mainly the N7 of guanine, O6 of Thymine, N3 of adenine etc [53,54,55]. Monofunctional reaction at the N7 of guanine has been implicated to a number of mutagens [56,57,58].

The computational data presented in Figure 5(i), 5(ii), 5(iii) and 5(iv) provide an intimate picture corresponding to the behavior of these compounds at the molecular level that led to the observed effect. Our studies show that only Bn1 and 2MP could form direct hydrogen bonding with the DNAs where the Bn1 binds at the wider minor groove (N2 of guanine) and to narrow minor groove (AT) [59] as well while 2MP binds only to the minor groove through O_2_ of thymine as most benzimidazole moiety do [60]. Both 2XP and 3BS failed to form any electrostatic linkages except via hydrophobic interaction and 2XP resided mainly in the narrow minor groove region of the nucleic acid while 3BS binds to the wider minor groove. 

The computational studies correspond well with the results of AMES test [61]. The AMES test enables the detection of potential binding to the GC sequences. GC rich sequences are normally found in the major groove and also in the wider minor groove region while the lack of significant mutagenic outcome suggests that this compound may target mainly the AT rich sequences, found largely in the minor groove region of the nucleic acid. The AMES analysis show that 3BS, like most alklating compounds [62], causes DNA mutation.

It thus appear in this study, equipping the benzyl-o-vanillin with additional benzyl-o-vanillin and benzimidazole clearly improved the anticancer activity of the benzyl-o-vanillin as seen in 2XP even though the interaction occurs only via hydrophobic interaction where a clear binding occurs mainly in the minor groove region of the DNA. The 3BS also showed an improved activity when compared to the parent compound (benzyl-o-vanillin) even though the interaction occurs only via hydrophobic interaction with the DNA but mainly in the wider minor groove region and the major groove. In addition, the presence of benzenesulfonate on 3BS structure gives this compound the advantage to alkylate the DNA via monofunctional alkylation reaction [63] and mutagenicity. Both compounds gave potent cytotoxic response towards the leukemia cell line although one compound binds in the wider minor groove and may be the major groove (3BS) and the other binds in the narrow minor groove region (2XP). However due to the position of binding and hydrophobic interaction with the guanines, 3BS showed mutagenic outcomes and 2XP on the contrary was anti-mutagenic. The higher cytotoxic activity by 3BS is most probably due to a more significant interference with DNA regulatory enzymes activity that tend to regulate their activity in the wider major groove like the DNAase and topoisomerases I [64], 2XP on the other hand, was slight less cytotoxic when compared to 3BS, and its principal site of DNA drug binding (minor groove) could have caused the apparent reduction in activity. The cytotoxic activity observed could have been due to the interaction of this compound with the TATA binding box (TATA binding protein) that are often bind to AT-rich sequence and governs a large number of transcription processes a pre-eminent example being the hypoxia responsive element alpha which governs the vascular endothelial growth factor (VEGF). The TATA sequence is commonly found in the minor groove region. This sequence is also important in the regulation of a variety of genes that governs mitochondrial functioning. The result of the caspase study shows a clear interaction of 2XP with the functioning of the mitochondria which further support this hypothesis.Our results show that the benzylvanillin alone have no strong anticancer activity even after it was combined with the benzimidazole, but after being disubstituted with another benzylvanaillin, stronger activity (4 times) was observed. Also, the combination of benzyloxy-3-methoxyphenyl with benzenesulfonate significantly improved the activity of 3BS as the presence of benzenesulfonate enabled it to have good alkylating activity. Hence, the present study provides a new insight of this novel benzylvanillin and benzimidazole derivatives serving as potential therapeutic agents against leukemia.

## Supporting Information

Figure S1 **Spectral results of DNA binding with Bn1, 2MP, 2XP and 3BS.** Titration UV-visible absorption spectra for addition of aliquots of calf thymus DNA solution to a 500µl buffered of, (i) Bn1 solution (20µM) at 25C°. The bathochromic shift induced by the drug is 0.17nm and the hypochromic effect is 5.3%, (ii) 2MP solution (20µM) at 25C°. The bathochromic shift induced upon the drug is 0.53nm and the hypochromic effect is 16.9%. Note the clear isosbestic behavior at -0.17 and -0.05 nm suggesting optical contributions from two distinct species. A wavelength of -0.17nm, showing maximal change because of free ligand depletion, was selected to monitor the DNA-binding process, (iii) 2XP solution (20µM) at 25C°. The bathochromic shift (rightward arrow) induced upon the drug is 3.97nm and the hypochromic effect is 60.4% (arrow downwards). Note the clear isosbestic behavior at -0.07 and 0.1nm suggesting optical contributions from two distinct species. A wavelength of -0.07nm, showing maximal change because of free ligand depletion, was selected to monitor the DNA-binding process, and (iv) 3BS solution (20µM) at 25C°. The bathochromic shift induced upon the drug is 5.36nm (rightwards arrow) and the hypochromic effect is 61.8% (arrow downwards). Note the clear isosbestic behavior at -0.1nm suggesting optical contributions from two distinct species. This wavelength showing maximal change because of free ligand depletion that occurs due to the DNA-binding process.
(TIF)
