# Supplementary figures and images for: Conjugation of Benzylvanillin and Benzimidazole Structure Improves DNA Binding with Enhanced Antileukemic Properties
Source: PLoS One. 2013 Nov 15;8(11):e80983. doi: 10.1371/journal.pone.0080983 (PMC3829952; doi:10.1371/journal.pone.0080983)

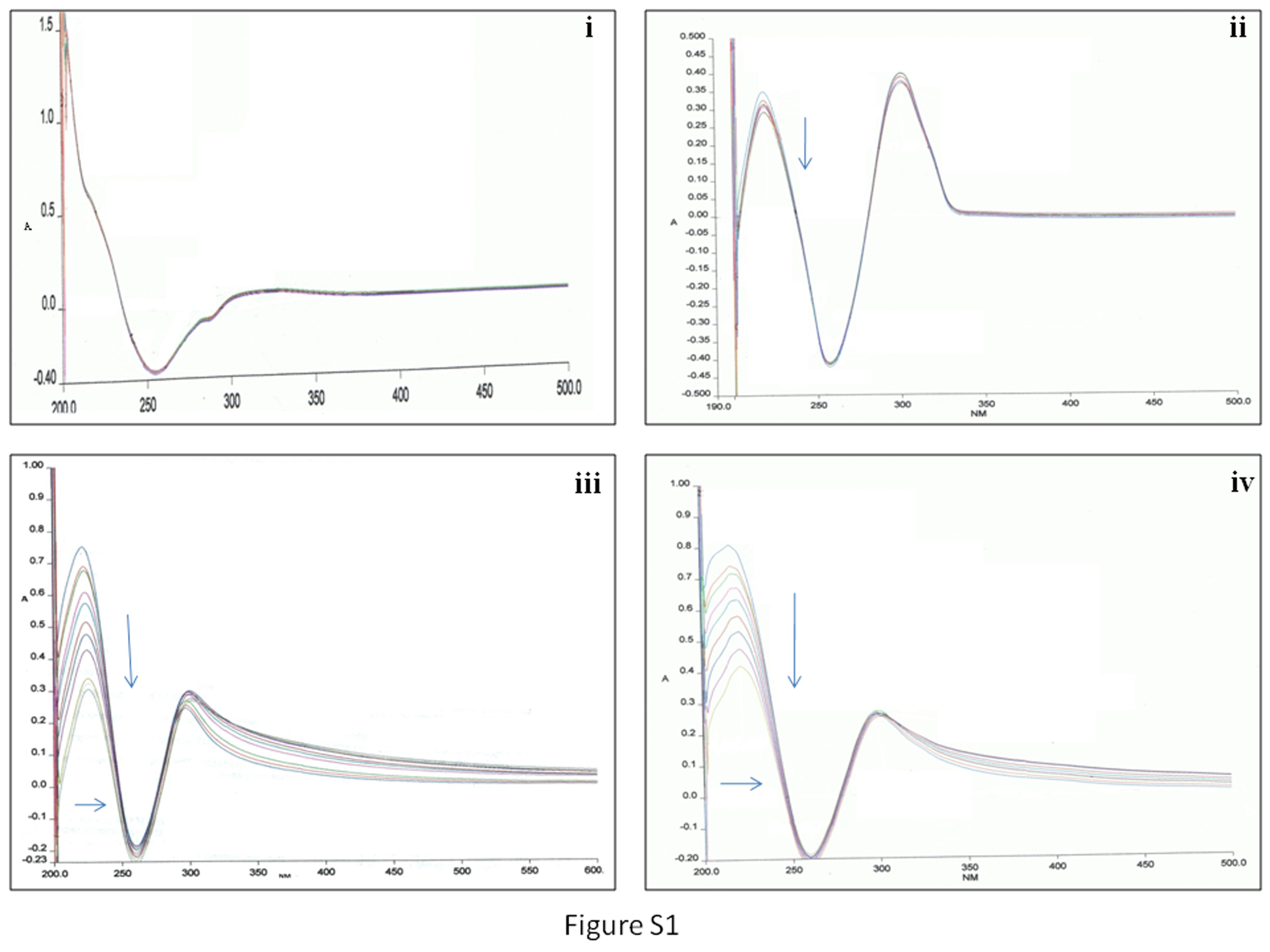

Supplement: Figure S1 — Spectral results of DNA binding with Bn1, 2MP, 2XP and 3BS. Titration UV-visible absorption spectra for addition of aliquots of calf thymus DNA solution to a 500µl buffered of, (i) Bn1 solution (20µM) at 25C°. The bathochromic shift induced by the drug is 0.17nm and the hypochromic effect is 5.3%, (ii) 2MP solution (20µM) at 25C°. The bathochromic shift induced upon the drug is 0.53nm and the hypochromic effect is 16.9%. Note the clear isosbestic behavior at -0.17 and -0.05 nm suggesting optical contributions from two distinct species. A wavelength of -0.17nm, showing maximal change because of free ligand depletion, was selected to monitor the DNA-binding process, (iii) 2XP solution (20µM) at 25C°. The bathochromic shift (rightward arrow) induced upon the drug is 3.97nm and the hypochromic effect is 60.4% (arrow downwards). Note the clear isosbestic behavior at -0.07 and 0.1nm suggesting optical contributions from two distinct species. A wavelength of -0.07nm, showing maximal change because of free ligand depletion, was selected to monitor the DNA-binding process, and (iv) 3BS solution (20µM) at 25C°. The bathochromic shift induced upon the drug is 5.36nm (rightwards arrow) and the hypochromic effect is 61.8% (arrow downwards). Note the clear isosbestic behavior at -0.1nm suggesting optical contributions from two distinct species. This wavelength showing maximal change because of free ligand depletion that occurs due to the DNA-binding process. (TIF) [file pone.0080983.s001.tif]
